# Supplementary material for: Human Papillomavirus Same Genotype Persistence and Risk of Cervical Intraepithelial Neoplasia2+ Recurrence
Source: Cancers (Basel). 2021 Jul 21;13(15):3664. doi: 10.3390/cancers13153664 (PMC8345074; doi:10.3390/cancers13153664)
Supplement: Supplementary file 1 [file cancers-13-03664-s001.zip › cancers-1288276-supplementary.pdf]

Supplementary Materials: Human Papillomavirus Same Genotype Persistence and Risk of Cervical Intraepithelial Neoplasia 2+ Recurrence

Anna Daniela Iacobone, Davide Radice, Maria Teresa Sandri, Eleonora Petra Preti, Maria Elena Guerrieri, Ailyn Mariela Vidal Urbinati, Ida Pino, Dorella Franchi, Rita Passerini and Fabio Bottari

Table S1. Transition probabilities at 3–9 months (first follow-up visit) by baseline infection status, *N* (row %).

Baseline	Follow-up		
	Negative	Single	Multiple
Single ($N = 296$)	236 (79.7)	55 (18.6)	5 (1.7)
Multiple ($N = 112$)	76 (67.9)	19 (17.0)	17 (15.2)

Symmetry test, $p = 0.001$.

Table S2. Transition probabilities at 3–9 months (first follow-up visit) by baseline number of infections, *N* (row %).

No. of infections at baseline	No. of infections at follow-up, N (row %)			
	Negative	1	2	≥3
1 ($N = 296$)	236 (79.7)	55 (18.6)	5 (1.7)	0
2 ($N = 83$)	60 (72.3)	14 (16.9)	9 (10.8)	0
≥3 ($N = 29$)	16 (55.2)	5 (17.2)	4 (13.8)	4 (13.8)

Symmetry test, $p = 0.001$.

Table S3. Infections status at baseline and at first follow-up visit by histology.

Histology	Baseline [a] *N* (column %)		Follow-up [b] *N* (column %)		
	Single $N = 296$	Multiple $N = 112$	Negative $N = 312$	Single $N = 74$	Multiple $N = 22$
CIN1	50 (16.9)	22 (19.4)	59 (18.9)	9 (12.2)	4 (18.2)
CIN2/3/AIS	227 (76.7)	86 (76.8)	232 (74.4)	63 (85.1)	18 (81.8)
ICC	19 (6.4)	4 (3.6)	21 (6.7)	2 (2.7)	0

Fisher's exact test, [a] $p = 0.49$; [b] $p = 0.30$.

Table S4. Cumulative Relapse Incidence at 2-years from first follow-up by HPV16 status in persistent patients with one HR genotype only ($N = 74$).

HPV 16 Status	Events/At risk	Cumulative Incidence (95% CI)	*p*-value [a]
Overall	14/74	26.2 (14.1-40.2)	-
HPV 16-/Other HR	5/32	24.8 (8.3-45.7)	
HPV 16+	9/42	28.5 (11.6-48.2)	0.68

[a] Gray's test.
